# Downregulation of amphiregulin improves cardiac hypertrophy via attenuating oxidative stress and apoptosis

**DOI:** 10.1186/s13062-022-00334-w

**Published:** 2022-08-22

**Authors:** Mingyue Ji, Yun Liu, Zhi Zuo, Cheng Xu, Li Lin, Yong Li

**Affiliations:** 1 Department of Cardiology, Lianshui County People’s Hospital, Huaian, China; 2 Department of Intensive Care Medicine, the First Affiliated Hospital of Nanjing Medical University, Nanjing, China; 3 Department of Cardiology, the First Affiliated Hospital of Nanjing Medical University, 300 Guangzhou Road, Nanjing, 210029 Jiangsu Province China; 4 Department of Cardiovascular Medicine, East Hospital, Tongji University School of Medicine, 150 JimoRoad, Shanghai, 200120 China

**Keywords:** Amphiregulin, Cardiac hypertrophy, Oxidative stress, Apoptosis

## Abstract

Amphiregulin (AREG) is a ligand of epidermal growth factor receptor and participates in the fibrosis of multiple organs. However, whether AREG can regulate hypertrophic cardiomyopathy is not well known. This research aims to explore the effect of AREG on cardiac hypertrophy, and whether the oxidative stress and apoptosis was involved in the influence of AREG on cardiac hypertrophy. Angiotensin (Ang) II induced cardiac hypertrophy in mice and neonatal rat cardiomyocytes (NRCMs) or HL-1 cells in vitro. AREG expressions raised in the heart of mice. After AREG downregulation, the increases of Ang II induced cardiac weight and cardiomyocytes area were inhibited. Down-regulation of AREG could inhibit Ang II induced the increases of atrial natriuretic peptide, brain natriuretic peptide, beta-myosin heavy chain in the heart of mice, and NRCMs and HL-1 cells. The enhancement of oxidative stress in mice heart with Ang II treatment was alleviated by AREG knockdown. The raises of Ang II induced Bax and cleaved caspase3 in mice heart were inhibited by AREG downregulation. AREG downregulation reduced myocardial hypertrophy via inhibition of oxidative and apoptosis. AREG may be a target for future cardiac hypertrophy treatment.

## Introduction

Myocardial hypertrophy can cause congestive heart failure, and is the main cause of incidence rate and mortality worldwide [1]. Cardiac hypertrophy is related to the adverse consequences of various states of cardiovascular disease. It is an important risk factor of cardiovascular disease, including heart failure, arrhythmia and sudden death [2]. Pathological cardiac hypertrophy begins with an adaptive response to increased workload. However, continuous hemodynamic pressure can lead to maladjustment and eventually lead to heart failure [3]. Increased cardiomyocyte size and heart weight [4–6], as well as increased expression of genes, such as atrial natriuretic peptide (ANP), brain natriuretic peptide (BNP) and beta-myosin heavy chain (β-MHC) are the features of cardiac hypertrophy [7].

Amphiregulin (AREG) is a ligand of epidermal growth factor receptor (EGFR), which is widely expressed in cardiomyocytes and fibroblasts [8]. Under physiological conditions, the activation of EGFR in the heart induces major intracellular signal cascades and control fibroblast proliferation, migration and collagen synthesis. However, under the chronic stress state characterized by the continuous increase of AREG, the long-term activation of EGFR can enhance the activation, proliferation, myofibroblast differentiation, migration and collagen synthesis of cardiac fibroblasts [9]. However, whether AREG participated in the regulation of cardiac hypertrophy is still unclear.

It is well known that oxidative stress promotes protein oxidation, lipid peroxidation, protease activation, DNA fragmentation and gene expression changes, resulting in cardiomyocyte damage and cardiomyocyte loss in patients with different types of heart disease [10]. Oxidative stress was enhanced in the disease of cardiac hypertrophy [11]. It is still unclear whether AREG modulate oxidative stress in hypertrophic cardiomyopathy.

Apoptosis, also known as programmed cell death, plays an important role in the physiological development and pathological process of a variety of cells and tissues. Increased diastolic function and decreased systolic function determine pathological hypertrophy, which usually causes heart failure and is related to increased cardiomyocyte death [12]. The phenotypic variability and heterogeneous remodeling observed in hypertrophic cardiomyopathy is the end-result of many factors to include apoptosis [13]. Angiotensin (Ang) II, a member of renin-angiotensin system, traditionally viewed as a regulator of apoptosis in cardiovascular system [14, 15]. The purpose of this research was to explore the role of AREG on cardiomyocytes apoptosis of hypertrophic cardiomyopathy. In summary, this present study would probe the influences of AREG on cardiac hypertrophy and fibrosis, and quest whether AREG regulates cardiac hypertrophy through affecting oxidative stress and apoptosis.

## Results

### Expression of AREG

**Fig. 1:**
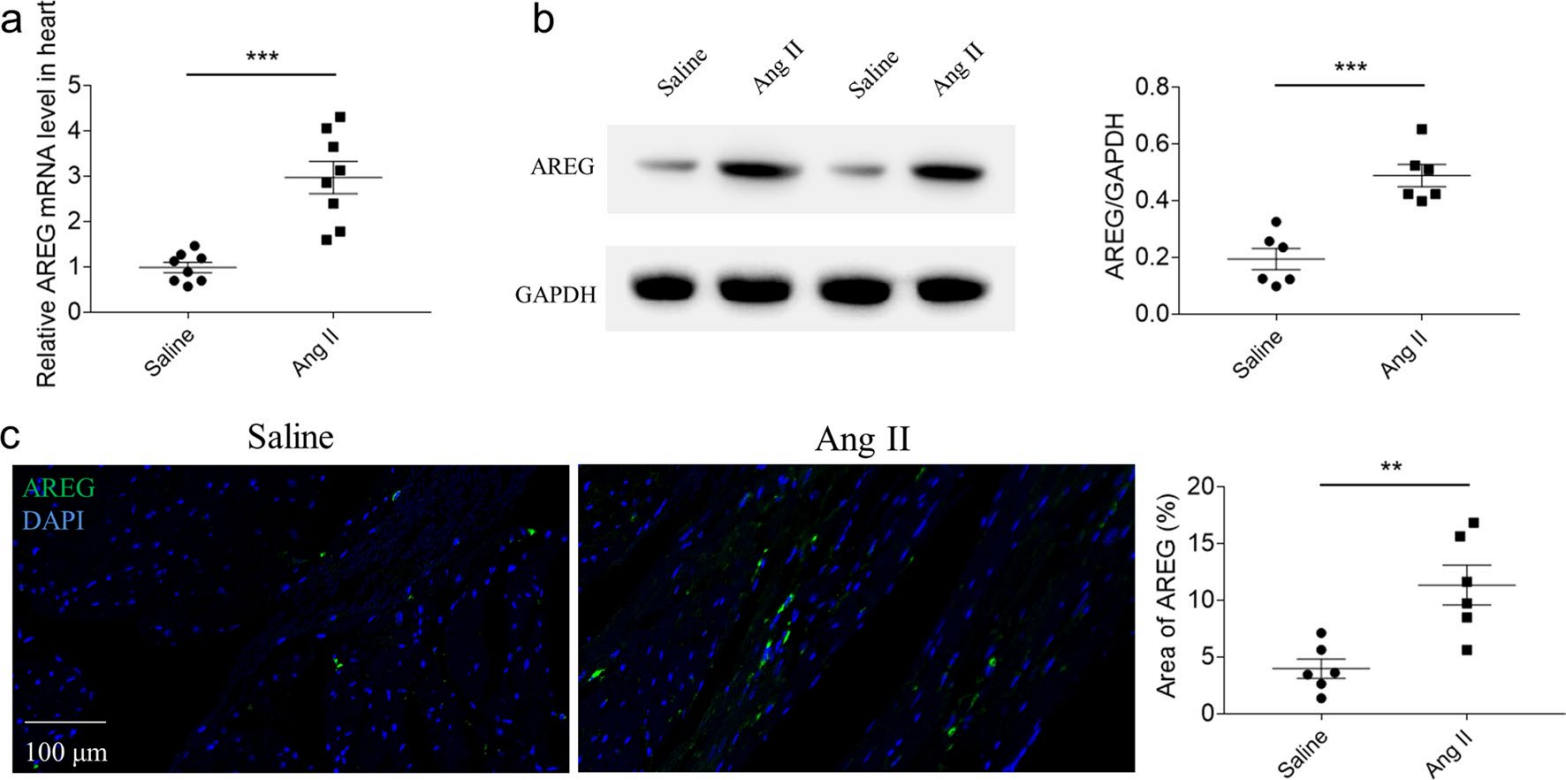
Expression of AREG. **a**, AREG mRNA level raised in mice heart with Ang II treatment. **b** and **c**, AREG protein level was raised in mice with Ang II treatment detected by western blotting (**b**) or immunofluorescence staining (**c**). *n* = 8 (**a**) or 6 (**b** and **c**) for each group. Express the results as mean ± SEM

AREG mRNA level raised in the mice heart treated with Ang II (Fig. 1a). AREG protein level also increased in the mice heart with Ang II treatment detected by western blotting (Fig. 1b) or immunofluorescence staining (Fig. 1c).

### AREG knockdown alleviated cardiac hypertrophy of mice

**Fig. 2:**
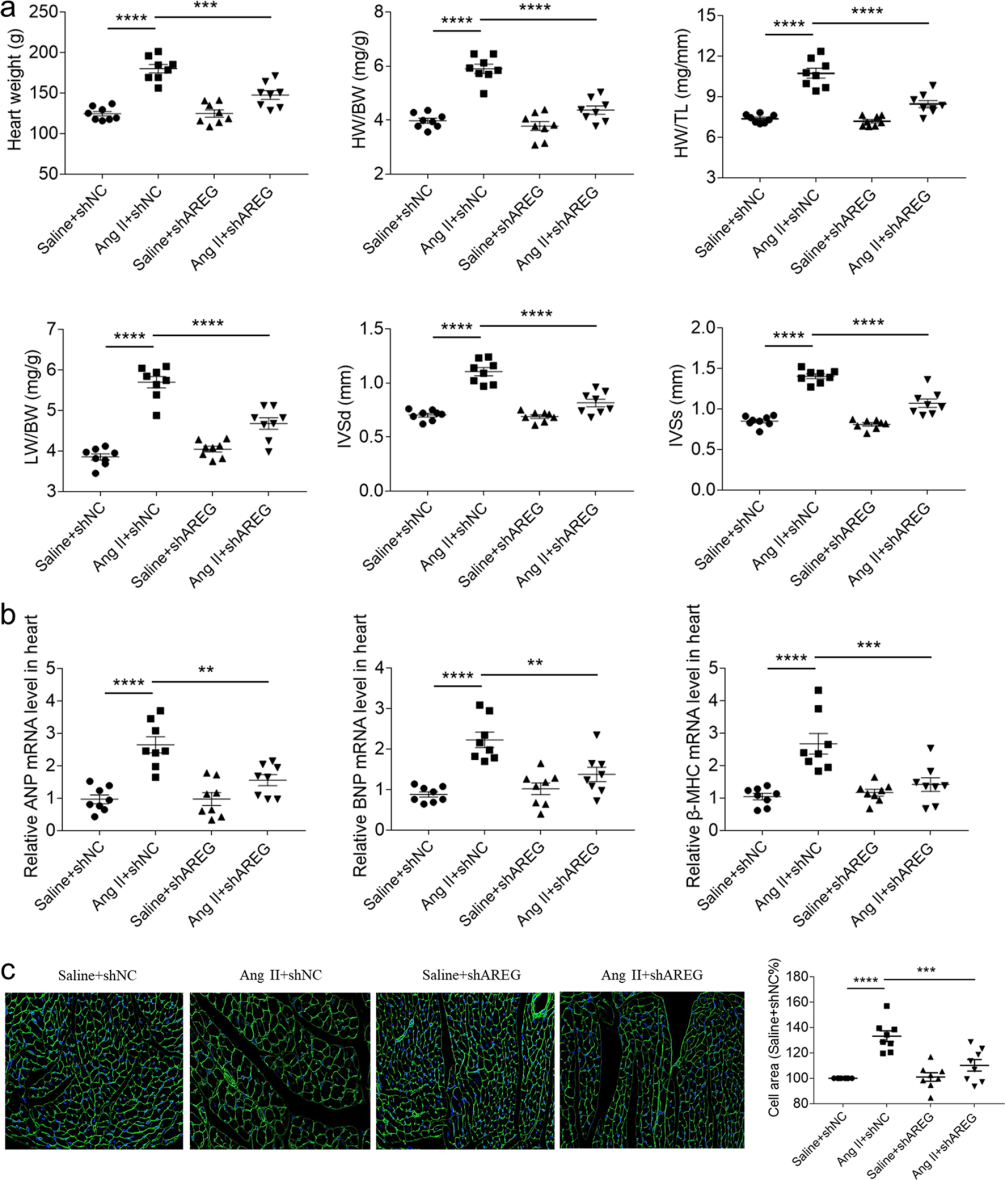
AREG knockdown alleviated cardiac hypertrophy of mice. **a**, The enhancements of Ang II induced heart weight, HW/BW, HW/TL, LW/BW, IVSd andIVSs of mice were alleviated by AREG downregulation. **b**, The enhancements of Ang II induced ANP, BNP and β-MHC in mice heart were alleviated by AREG downregulation. **c**, The enhancement of Ang II induced cardiomyocytes area of mice was alleviated by AREG downregulation. *n* = 8 for each group. Express the results as mean ± SEM

Ang II administration increased heart weight (HW), HW/body weight (BW), HW/tibial length (TL), left ventricle weight (LW)/BW, interventricular septal thickness at end diastole (IVSd) and interventricular septal thickness at end systole (IVSs) of mice, which were inhibited by AREG knockdown (Fig. 2a). The levels of ANP, BNP and β-MHC were elevated in mice heart with Ang II treatment. These increases were suppressed after downregulation of AREG (Fig. 2b). The area of cardiomyocytes raised in mice with Ang II treatment, and was attenuated by AREG knockdown (Fig. 2c).

### AREG knockdown alleviated cardiac fibrosis of mice

**Fig. 3:**
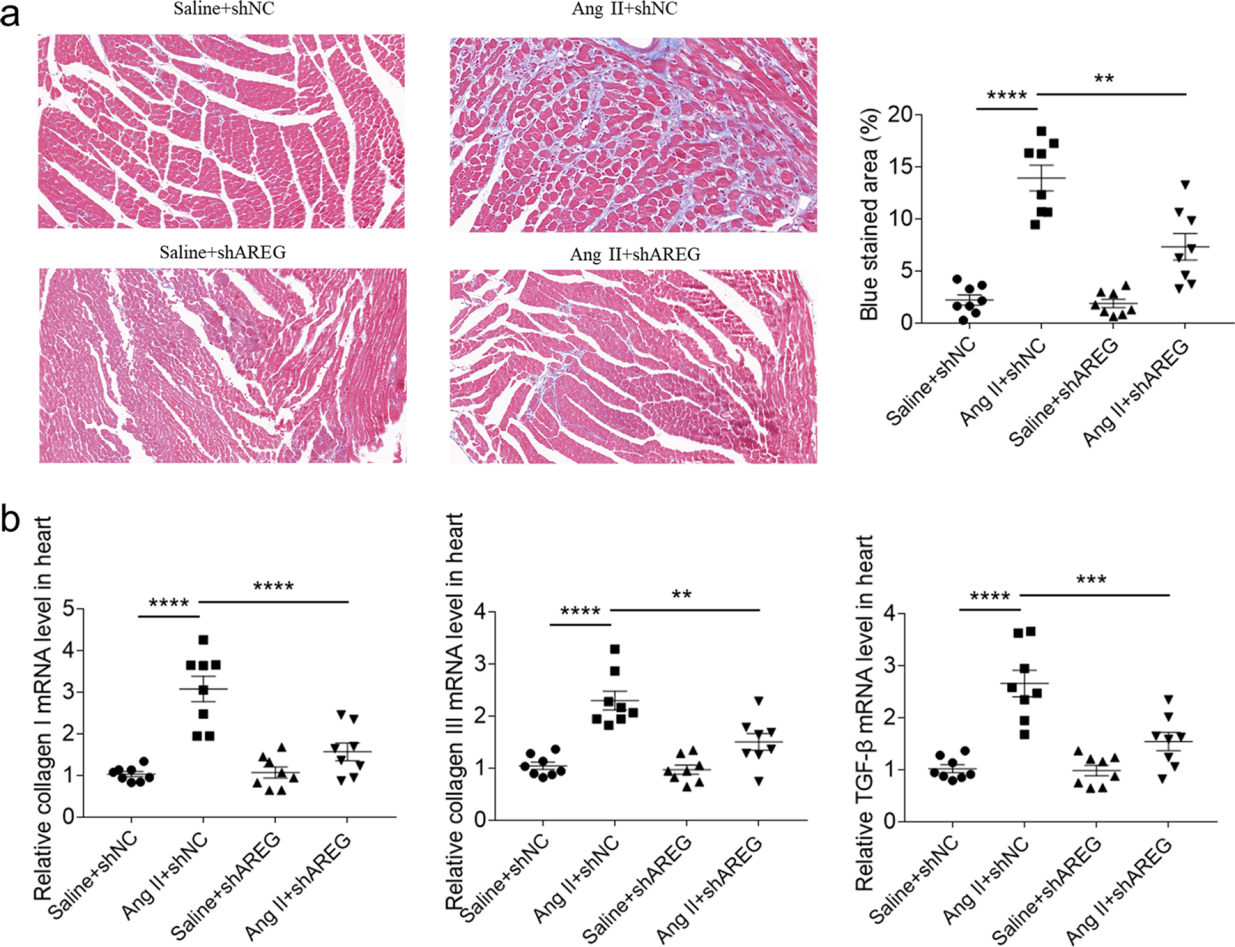
AREG knockdown alleviated cardiac fibrosis of mice. **a**, The Ang II induced cardiac fibrosis of mice was alleviated by AREG downregulation. **b**, The enhancements of Ang II induced collagen I, collagen III and TGF-β in mice heart were alleviated by AREG downregulation. *n* = 8 for each group. Express the results as mean ± SEM

Cardiac fibrosis was detected by masson staining (blue). AREG knockout can reduce AngIIinduced myocardial fibrosis in mice (Fig. 3a). The collagen I, collagen III and TGF-β levels raised in mice heart with Ang II treatment, which were inhibited after AREG downregulation (Fig. 3b).

### Effects of AREG on cardiomyocytes hypertrophy

**Fig. 4:**
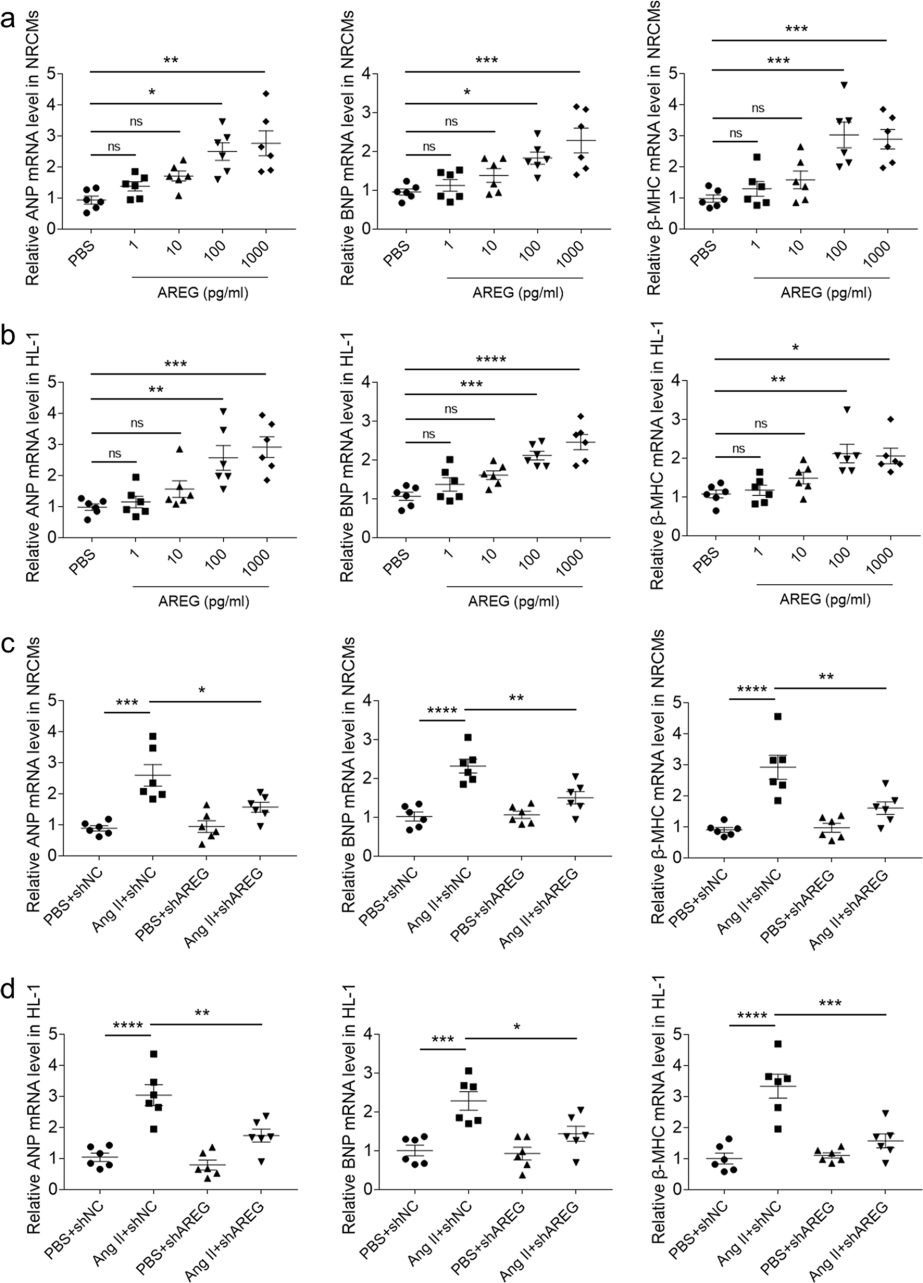
Effects of AREG on cardiomyocytes hypertrophy. **a**, The ANP, BNP and β-MHC levels raised in the NRCMs after treating with AREG. **b**, The ANP, BNP and β-MHC levels raised in the HL-1 cells after treating with AREG. **c**, The enhancemnets of Ang II induced ANP, BNP and β-MHC in the NRCMs were alleviated by AREG downregulation. **d**, The enhancements of Ang II induced ANP, BNP and β-MHC in the HL-1 cells were alleviated by AREG downregulation. *n* = 6 for each group. Express the results as mean ± SEM

Four doses of AREG were used to search the influence of AREG on cardiomyocytes hypertrophy. Neonatal rat cardiomyocytes (NRCMs) was treated with Ang II to induce hypertrophic model [16, 17]. The doses of 1 and 10 ng/ml have no effect on the hypertrophy of NRCMs. The doses of 100 and 1000 ng/ml enhanced the expressions of ANP, BNP and β-MHC in NRCMs (Fig. 4a). The doses of 1 and 10 ng/ml have no effect on the hypertrophy of HL-1 cells. The doses of 100 and 1000 ng/ml enhanced the expressions of ANP, BNP and β-MHC in HL-1 cells (Fig. 4b). The findings indicated that AREG induced cardiomyocytes hypertrophy. Knockdown of AREG restrained the enhancements of Ang II induced ANP, BNP and β-MHC in NRCMs (Fig. 4c). The enhancements of Ang II induced ANP, BNP and β-MHC in HL-1 were also suppressed after AREG knockdown (Fig. 4d).

### Effects of AREG on oxidative stress

**Fig. 5:**
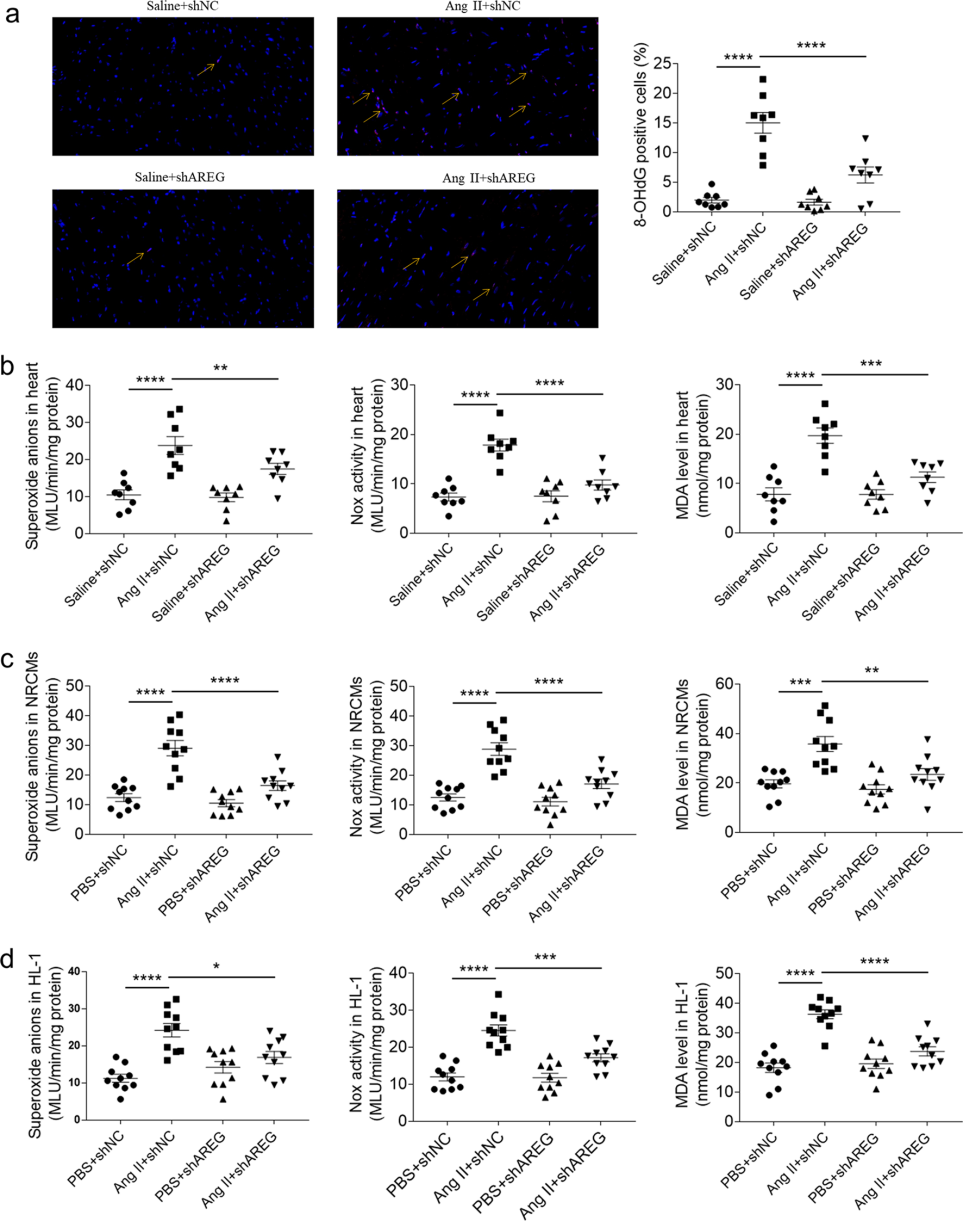
Effects of AREG on oxidative stress. **a**, The increase of Ang II induced 8-OHdG positive cells in mice heart was alleviated by AREG downregulation. Magnification 400X. **b**, The enhancements of Ang II induced superoxide anions, Nox activity and MDA in mice heart were alleviated by AREG downregulation. **c**, The enhancements of Ang II induced superoxide anions, Nox activity and MDA in the NRCMs were alleviated by AREG downregulation. **d**, The enhancements of Ang II induced superoxide anions, Nox activity and MDA in the HL-1 cells were alleviated by AREG downregulation. *n* = 8 (**a** and **b**) or 10 (**c** and **d**) for each group. Express the results as mean ± SEM. Magnification

The 8-hydroxy-2′ -deoxyguanosine (8-OHdG) positive cells number was increased in mice heart with Ang II treatment, and was suppressed after AREG knockdown (Fig. 5a). The superoxide anions, NADPH oxidase (Nox) activity and malondialdehyde (MDA) levels raised in mice heart with Ang II treatment, and were attenuated by AREG knockdown (Fig. 5b). Ang II elevated the superoxide anions, Nox activity and MDA levels in NRCMs, and were reversed after AREG downregulation (Fig. 5c). Downregulating of AREG inhibited the enhancements of superoxide anions, Nox activity and MDA induced by Ang II in the HL-1 cells (Fig. 5d).

### Effects of AREG on apoptosis

**Fig. 6:**
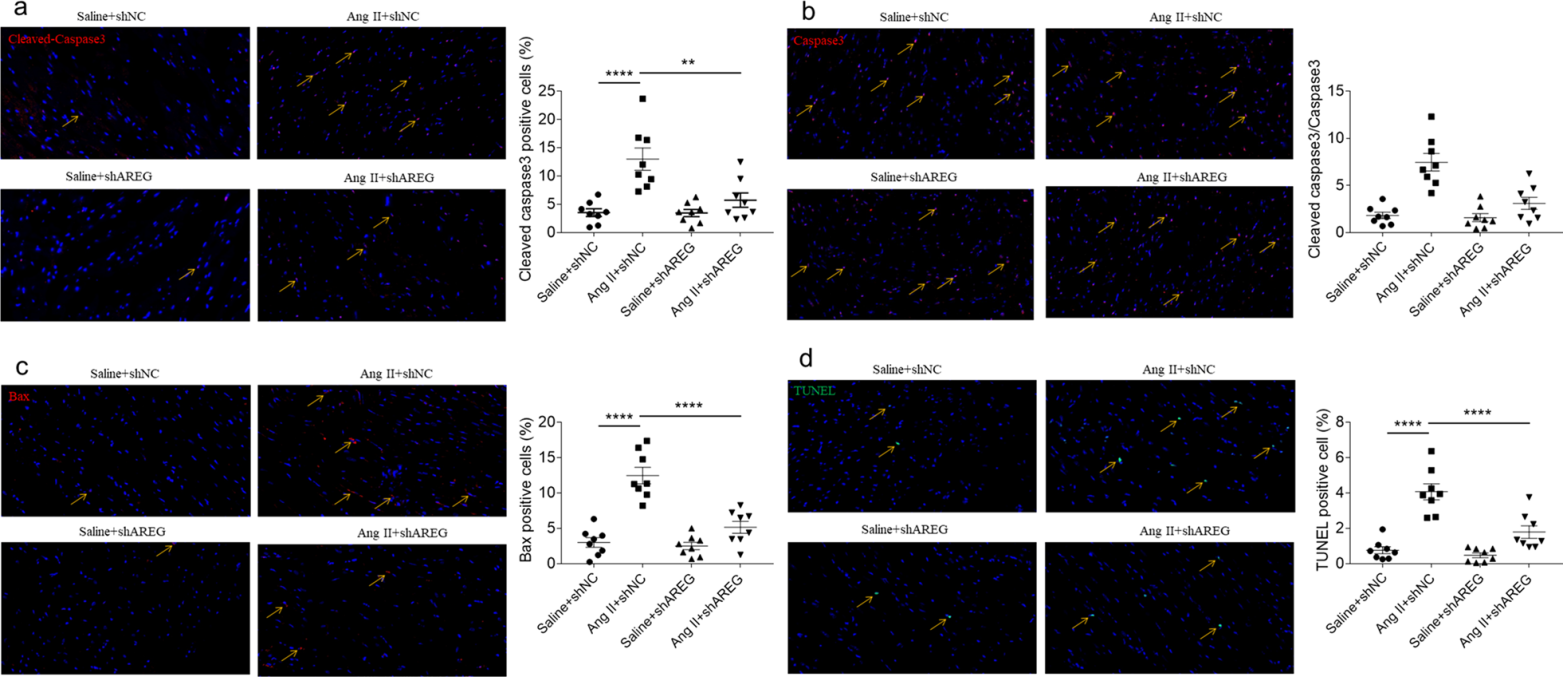
Effects of AREG on apoptosis. **a**, The enhancement of Ang II induced cleaved caspase3 positive cells in mice heart was alleviated by AREG downregulation. **b**, The enhancement of Ang II induced cleaved caspase3/caspase3 in mice heart was alleviated by AREG downregulation. **c**, The enhancement of Ang II induced Bax positive cells in mice heart was alleviated by AREG downregulation. **d**, The enhancement of Ang II induced TUNEL positive cells in mice heart was alleviated by AREG downregulation. *n* = 8 for each group. Express the results as mean ± SEM. Magnification 400X

The number of cleaved caspase3 positive cell raised in mice heart with Ang II treatment, which was suppressed after AREG knockdown (Fig. 6a). The level of caspase3/caspase3 was increased in the heart of Ang II-treated mice, and this increase was suppressed by AREG knockdown (Fig. 6b). The Bax positive cell number raised in mice heart with Ang II treatment, which was attenuated by AREG knockdown (Fig. 6c). The TUNEL positive cell number raised in mice heart with Ang II treatment, which was inhibited after AREG knockdown (Fig. 6d).

## Discussion

It was found that AREG expression enhanced in mice heart with Ang II treatment. Knockdown of AREG alleviated cardiac hypertrophy and fibrosis in mice heart with Ang II treatment. AREG downregulation attenuated the increases of oxidative stress and apoptosis induced by Ang II in mice heart.

AREG is a member of epidermal growth factor family, which plays a key role in cardiovascular diseases [18]. AREG expression is upregulated in many disease models. For example, AREG expression was significantly enhanced in acute lung injury induced by lipopolysaccharide and pulmonary fibrosis induced by bleomycin [19, 20]. AREG expression also raised in MI mice heart [9]. We present found that AREG expression raised in mice heart with Ang II treatment. In addition, AREG expressions were also elevated in NRCMs and HL-1 cells treated with Ang II. These outcomes showed that the level of AREG increased in hypertrophic cardiomyopathy. It remained to be further explored that AREG may be involved in the regulation of myocardial hypertrophy.

Previous researches have also showed that AREG was involved in several organ fibrosis diseases such as lung and liver fibrosis [21, 22]. Similarly, AREG administration enhanced fibrosis of heart, while AREG knockdown relieved cardiac fibrosis of myocardial infarction [9, 23]. Our current results showed that AREG knockdown alleviated Ang II induced cardiac fibrosis in hypertrophic cardiomyopathy, which supported the influence of AREG on promoting cardiac fibrosis reported as previous studies. We further found that the enhancements of Ang II induced heart weight and cardiomyocytes area were attenuated after downregulation of AREG. Moreover, the ANP, BNP and β-MHC expressions raised in the NRCMs and HL-1 cells after AREG administration, and the enhancements of Ang II induced ANP, BNP and β-MHC in the NRCMs and HL-1 cells were suppressed by AREG knockdown. Our outcomes demonstrated that AREG enhanced cardiac hypertrophy, and targeting of AREG could alleviate hypertrophic cardiomyopathy.

Oxidative stress is the accumulation of free radicals due to the imbalance between the production of reactive oxygen species and antioxidant defense. In the heart, reactive oxygen species activate signaling pathways involved in cardiomyocyte hypertrophy and interstitial fibrosis [24]. NADPH oxidases are transmembrane enzymes, which play an important role in mediating cardiac dysfunction by transferring electrons from NADPH to molecular oxygen to produce superoxide and promote superoxide production. [25]. MDA and 8-OHdG have been widely used as biomarkers of oxidative stress [26, 27]. Cardiac hypertrophy may contribute to heart failure and is closely related to oxidative stress [28]. In current research, we found that AREG knockdown significantly weakened the enhancement of Ang II induced 8-OHdG positive cells in mice heart. The increases of Ang II induced superoxide anions, Nox activity and MDA in mice heart and cultured cardiomyocytes were suppressed after downregulating of AREG. These outcomes showed that AREG downregulation weakened cardiac hypertrophy via attenuation of oxidative stress.

In physiology, apoptosis plays an important role in maintaining homeostasis through destroying unnecessary cells. However, excessive apoptosis can lead to pathological state and eventually organ failure. The raise of cardiac oxidative stress plays a key role in mediating cardiac hypertrophy, fibrosis and apoptosis, as well as heart failure. [29]. Cardiomyocyte apoptosis raised in hypertrophic cardiomyopathy of mice, and this increase resulted in fibrosis interstitial fibrosis at later stages [30]. We present found that the increases of Ang II induced Bax, cleaved caspase3 and TUNEL positive cells in mice heart were inhibited by downregulation of AREG. These results indicated that AREG downregulation mitigated the apoptosis to improve hypertrophic cardiomyopathy.

In conclusions, the AREG expression raised in the heart of hypertrophy. The down-regulation of AREG could reduce myocardial hypertrophy by reducing oxidative stress and apoptosis. AREG targeted therapy may be a strategy for hypertrophic cardiomyopathy in the future.

## Materials and methods

### Animals and treatment

Kept the 8–10 week old male C57/BL6/J mice (Vital River Biological Co., Ltd, Beijing, China) in a 12 h-light dark cycle, and was free to standard chow and tap water. All experiments were approved by the Experimental Animal Care and Use Committee of Nanjing Medical University (Nanjing, China) and followed the Guide for the Care and Use of Laboratory Animals (NIH publication No. 85-23, 1996). With osmotic minipumps (model 2004; ALZET, CA, USA) surgically placed below the neck, the mice were infused with Ang II (1.44 mg/kg/day, Sigma, MO, USA) or saline (solvent control) at a rate of 0.25 μl/h for four weeks. Meanwhile, AREG was downregulated by adeno-associated virus (shAREG) injection via tail vein (2 * 10^11^ in 200 μl saline; OBIO, Shanghai, China). shNC was used as a control.

### Echocardiography

Four weeks after Ang II infusion, transthoracic echocardiography was performed under isoflurane (1.5–2.0%) anesthesia using an ultrasound system (VisualSonics, Toronto, Canada) with a 21 MHz probe. The mean value of three consecutive cardiac cycles was measured. Measured the LW, IVSd and IVSs. After echocardiography, the mice were sacrificed and the HW, HW/ BW, HW/ TL and LW/BW were measured.

### Wheat germ agglutinin staining

The heart samples were collected via euthanasia after echocardiography. Stained the heart sections with FITC-conjugated wheat germ agglutinin (WGA; Invitrogen Inc., CA, USA) to measure the cross-sectional area of cardiomyocytes. Selected three to five random fields (around 30–50 cells per field) from three slices of each animal, observed under confocal microscope (Carl Zeiss GmbH, Oberkochen, Germany) and analyzed with Zeiss software.

### Masson staining

Using Masson staining method (Service Biological Technology Co., Ltd, Wuhan, China) to observe the 5 µm thick heart section and determine the degree of cardiac fibrosis (blue). Obtained the tissue sections from the mouse heart with a light microscope (Carl Zeiss GmbH, Oberkochen, Germany). Analyzed images with Image-Pro Plus software (Media Cybernetics, Inc., MD, USA).

### Culture of cardiomyocytes isolated from neonatal rat and treatment

NRCMs were isolated from neonatal Sprague Dawley rats aged 1–2 days (Vital River Biological Co.). Hearts were removed and digested in PBS containing type II collagenase (Worthington Biochemical Corp., NJ, USA) and trypsin (Sigma, MO, USA). Discarded the atria and great vessels. Cut the ventricles into small pieces and digested with type II collagenase and trypsin. In order to reduce fibroblasts and enrich for cardiomyocytes, collected and cultured cells from digestion in Complete Dulbecco's modified Eagle's medium (DMEM; GIBCO, Invitrogen Inc.) for 2–4 h. Cultured the cardiomyocytes with 5% CO_2_ at 37 °C. Firstly, divided the NRCMs into PBS and four doses of AREG groups (1, 10, 100 and 1000 pg/ml; R&D, Shanghai, China) for 24 h. Secondly, NRCMs were treated with adenovirus-control (shNC) or adenovirus-mediated AREG knockdown (shAREG; Genechem, Shanghai, China). Meanwhile, Ang II (10^–6^ M) was added into the medium for 24 h.

### Culture of HL-1 cells and treatment

Mice cardiomyocytes HL-1 cells were cultured in intact Dulbecco modified Eagle medium, and 10% fetal bovine serum (Nanjing Bio-Channel Biotechnology Co., Ltd, Nanjing, China) was added at 37 °C with 5% CO_2_ and 95% air. Treated HL-1 cells the same as NRCMs.

### Quantitative real-time PCR

Used quantitative real-time PCR (qRT-PCR) to determine the mRNA level. Isolated total RNA with Trizol reagent (Invitrogen, USA). Obtained the mRNA concentration with NANODROP ONE (Thermo, Shanghai, China), and 0.5 μg of total RNA was reverse transcribed to cDNA with 10 μl of random primers and PrimeScript™ RT Master mix (Takara Biotechnology Co., Ltd.). Performed qRT-PCR with the ABI Prism 7900 sequence detection system (Applied Biosystems, CA, USA). The primers were shown in Table 1. All samples were amplified three times in 384-well plates. The mean Ct value was normalized to endogenous control (GAPDH) values and then calculated Δcycle threshold (ΔCT) value to determine the relative mRNA expression level. Then calculated the 2^−ΔΔCt^ values.

**Table 1:** Primers used for qRT-PCR

Gene	Species	Forward primer	Reverse primer
Collagen I	Mouse	AAGAAGACATCCCTGAAGTCA	TTGTGGCAGATACAGATCAAG
Collagen III	Mouse	TTGGGATGCAGCCACCTTG	CGCAAAGGACAGATCCTGAG
TGF-β	Mouse	CGCAACAACGCCATCTATGA	ACTGCTTCCCGAATGTCTGA
ANP	Mouse	CCTAAGCCCTTGTGGTGTGT	CAGAGTGGGAGAGGCAAGAC
BNP	Mouse	AGACCCAGGCAGAGTCAGAA	CAGCTCTTGAAGGACCAAGG
β-MHC	Mouse	CTTCAACCACCACATGTTCG	TCTCGATGAGGTCAATGCAG
GAPDH	Mouse	CCTTCCGTGTTCCTACCCC	GCCCAAGATGCCCTTCAGT
ANP	Rat	GAGCAAATCCCGTATACAGTGC	ATCTTCTACCGGCATCTCCTCC
BNP	Rat	GCTGCTGGAGCTGATAAGAGAA	GTTCTTTTGTAGGGCCTTGGTC
β-MHC	Rat	GCTGCTGGAGCTGATAAGAGAA	GTTCTTTTGTAGGGCCTTGGTC
GAPDH	Rat	GGCACAGTCAAGGCTGAGAATG	ATGGTGGTGAAGACGCCAGTA

### Western blotting

Total protein from heart and cell samples was extracted in RIPA lysis buffer and their concentrations were measured by the BCA method (Beyotime Biotechnology, Shanghai, China). The protein samples were electrophoresed and then transferred to PVDF membranes. After blocking the non-specific antigen in 5% skim milk, immunoblotted membranes with primary antibodies of AREG reacted with mouse (Santa, Shnaghai, China) or GAPDH (Abcam). Band exposure was achieved by chemiluminescence.

### Immunofluorescence

Fixed the heart samples with 4% paraformaldehyde 24 h at room temperature. Afterwards, incubated the samples with the primary antibody against AREG (Santa, TX, USA), Bax (Abcam), cleaved caspase3 (CST, MA, USA), caspase3 (CST) or 8-OHdG (Santa) a whole night at 4 °C, and then incubated with the corresponding secondary antibody (Jackson ImmunoResearch, PA, USA) at room temperature for 2 h. Then, the nucleus were counterstained with 4’,6-diamidino-2-phenylindole (DAPI; Life Technologies Co., Grand Island, NY, USA). Took the images of fluorescent cell with a fluorescence microscope (Carl Zeiss GmbH, Oberkochen, Germany).

### Measurement of superoxide anions

Measured superoxide anion levels of the samples with fluorescein derived chemiluminescence. Detected the protein consistence with a BCA kit (Beyotime Biotechnology). Added dark adapted fluorescein (5 μM) to each sample to generate photon emission, and measured it once a minute with a microplate reader (BioTek) for 10 min. The level of superoxide anion was expressed as mean light units (MLU) per milligram per minute.

### Measurement of NADPH oxidase activity

Measured the activity of Nox in the sample with enhanced fluorescein chemiluminescence. As a substrate, NADPH (100 μM) was added into the media, and reacted with Nox to generate superoxide anions. Determined the light emissionproduced by the reaction of lucigenin (5 μM) and superoxide anions with a microplate reader (BioTek) once a minute for 10 min. The Nox activity level was presented as the MLU per milligram protein per minute.

### Determination of MDA level

Homogenized the samples in cracking buffer (Thermo Fisher Scientific, MA, USA). The protein concentration was detected by a BCA kit (Beyotime Biotechnology). The level of MDA was measured by ELISA kit (USCN Business Co., Ltd., Wuhan, China).

### Statistical analyses

Data were presented as mean ± SEM. The statistical significance between groups was assessed by one-way analysis of variance (ANOVA) of Bonferroni posttest using GraphPad Prism 6.0 (GraphPad software Inc., CA, USA), and a two-tailed *P*-value < 0.05 was defined as statistically significant.

## Data Availability

Available upon requests.

## Abbreviations

8-OHdG: 8-Hydroxy-2′ –deoxyguanosine
ANP: Atrial natriuretic peptide
AREG: Amphiregulin
BNP: Brain natriuretic peptide
β-MHC: Beta-myosin heavy chain
BW: Body weight
HW: Heart weight
IVSd: Interventricular septal thickness at end diastole
IVSs: Interventricular septal thickness at end systole
LW: Left ventricle weight
MDA: Malondialdehyde
Nox: NADPH oxidase
NRCMs: Neonatal rat cardiomyocytes
TL: Tibial length
